# Postmenopausal intestinal obstructive endometriosis: case report and review of the literature

**DOI:** 10.1590/S1516-31802008000300010

**Published:** 2008-05-01

**Authors:** Pedro Popoutchi, Carlos Renato dos Reis Lemos, Julio César Rosa e Silva, Antônio Alberto Nogueira, Omar Feres, José Joaquim Ribeiro da Rocha

**Keywords:** Intestines, Endometriosis, Surgery, Diagnosis, Postmenopause, Intestinos, Endometriose, Cirurgia, Diagnóstico, Pós-menopausa

## Abstract

**CONTEXT:**

Endometriosis is characterized by the presence of endometrial tissue outside the uterine cavity, which is commonly detected in gynecological practice but rarely reported as a coloproctological disorder. The objective of the present report was to discuss a rare case of postmenopausal intestinal endometriosis simulating a malignant lesion, following a review of the literature.

**CASE REPORT:**

A 74-year-old woman with complaints of hematochezia and tenesmus of two months’ duration accompanied by liquid feces and pelvic pain, but with no other gastrointestinal or gynecological complaints, was referred to our service. She had been menopausal for 22 years, with no hormone replacement treatment, and had undergone panhysterectomy three years before the referral to us, due to endometrial thickening and a right adnexal cyst. Five months before this referral, she had undergone laparotomy due to acute obstructive abdomen, which revealed a tumor mass involving the small bowel. Anatomopathological examination of the enterectomy suggested a hypothesis of intestinal endometriosis. A proctological examination was normal. Computed tomography of the pelvis revealed thickening of the rectosigmoid transition and colonoscopy revealed friable tumor formation in the rectum. A biopsy of the lesion revealed mucosal fragments of endometrial type, which led to a review of the previous anatomopathological examination. The patient underwent rectosigmoidectomy with protective transversotomy, with a good postoperative course, and anatomical examination confirmed the intestinal endometriosis. The patient subsequently suffered a stenosing recurrence of the lesion and has undergone colostomy since then.

## RESUMO

**CONTEXTO:**

A endometriose caracteriza-se pela presença de tecido endometrial fora da cavidade uterina, e a etiopatogenia ainda apresenta controvérsias. O objetivo desta publicação é apresentar e discutir, após revisão da literatura, um raro caso de endometriose intestinal na pós-menopausa que simulava uma lesão maligna.

**RELATO DE CASO:**

Mulher de 74 anos apresentou-se com queixas de hematoquezia e tenesmo há dois meses. Relatou também aparecimento de fezes líquidas e dor pélvica no mesmo período, negando outras queixas gastrointestinais ou ginecológicas. Como antecedentes de interesse, revelou que era menopausada há 22 anos, sem terapia de reposição hormonal e realizou uma pan-histerectomia há três anos por espessamento endometrial e cisto anexial direito. Há cinco meses foi submetida a laparotomia exploradora por abdome agudo obstrutivo, com o achado de uma massa tumoral envolvendo alças de delgado. O exame anatomopatológico da enterectomia sugeriu a hipótese de endometriose intestinal. O exame proctológico era normal. A tomografia computadorizada da pelve mostrou um espessamento da transição retossigmóide e a colonoscopia, uma tumoração friável e estenosante no reto alto. A biópsia da lesão revelou fragmentos de mucosa tipo endometrial, que motivou a revisão do anatomopatológico anterior. A paciente foi submetida a retossigmoidectomia abdominal com transversostomia protetora, tendo boa evolução no pós-operatório. O anatomopatológico confirmou endometriose intestinal. Evoluiu com recidiva estenosante da lesão e pemanece colostomizada desde então.

## INTRODUCTION

Endometriosis is an estrogen-dependent disease that usually occurs in women during the menacme. Its etiopathogenesis is still a matter of controversy.^1-3^ However, occurrences of endometriosis in patients with no menstrual flow,^4,5^ or the presence of lesions at sites where there is no direct contact with menstrual flow, such as the lungs and intestine,^6,7^ raise the hypothesis that metaplasia may develop in these areas and/or vascular transport may occur, especially if estrogen is not present.

The incidence of intestinal endometriosis ranges from 3 to 34%,^8-10^ and the sigmoid and rectum are more commonly involved. This disorder is more frequent among women during the menacme^6^ and its importance lies mainly in the need for a differential diagnosis with colon adenocarcinoma, which is the third most common type of cancer diagnosed in women.^11^

The objective of the present report was to describe and discuss a rare case of postmenopausal intestinal endometriosis simulating a malignant lesion, with emphasis on diagnostic and therapeutic methods, following a review of the literature.

## CASE REPORT

A 74-year-old white woman from Itabuna, State of Bahia, Brazil, sought the Coloproctology Service of our institution with complaints of hematochezia and liquid feces of two months’ duration, as well as tenesmus and pelvic pain.

Her antecedents of interest included eight pregnancies, two abortions, six vaginal deliveries, menopause of 22 years’ duration, presence of diabetes and hypertension, and no hormone replacement treatment. She reached the menarche at 13 years of age, and did not have any history of dysmenorrhea. She had undergone hysterectomy plus bilateral salpingo-oophorectomy three years before her referral to our service, due to endometrial thickening, with a postoperative anatomopathological diagnosis of endometrial glandular hyperplasia and discrete cellular atypia. On the same occasion, a mass of chocolate-colored content that occluded the upper third of the vagina and a paratubal cyst also containing chocolate-colored matter had been detected intraoperatively. Histological analysis led to a diagnosis of hematosalpinx with hemosiderin on the left side.

Five months before before her referral to our service, she was attended at the emergency service of Hospital das Clínicas, Faculdade de Medicina de Ribeirão Preto, Universidade de São Paulo (HC/FMRP/USP) with signs and symptoms of intestinal obstruction. She underwent exploratory laparotomy, which revealed a tumor mass surrounding some loops of the small intestine. Anatomopathological examination confirmed the presence of intestinal endometriosis.

**Figure 1:**
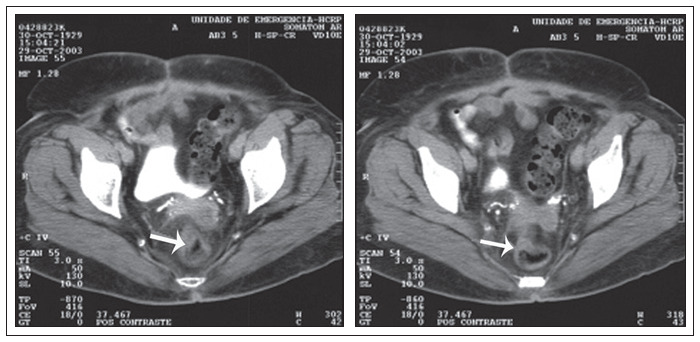
Computed tomography of the abdomen and pelvis of a 74-years-old woman, showing parietal thickening in the rectosigmoid transition.

**Figure 2:**
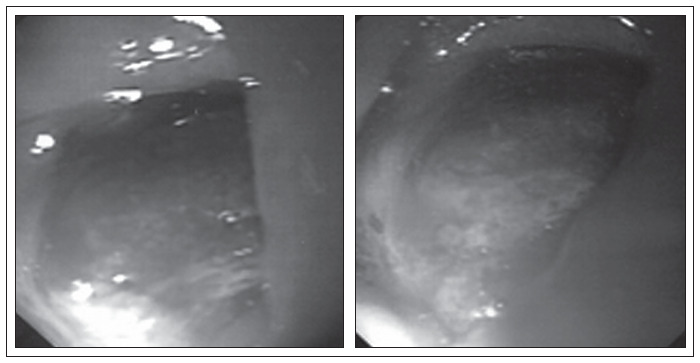
Preoperative colonoscopy, showing friable and stenosing tumor formation in the upper rectum.

Physical and proctological examinations gave normal results, except for grade II obesity. Colonoscopy was requested (Figure 1), which revealed a friable and stenosing tumor formation in the upper rectum. A biopsy revealed mucosal fragments of endometrial type. Tomography of the pelvis (Figure 2) only showed parietal thickening in the rectosigmoid transition.

**Figure 3:**
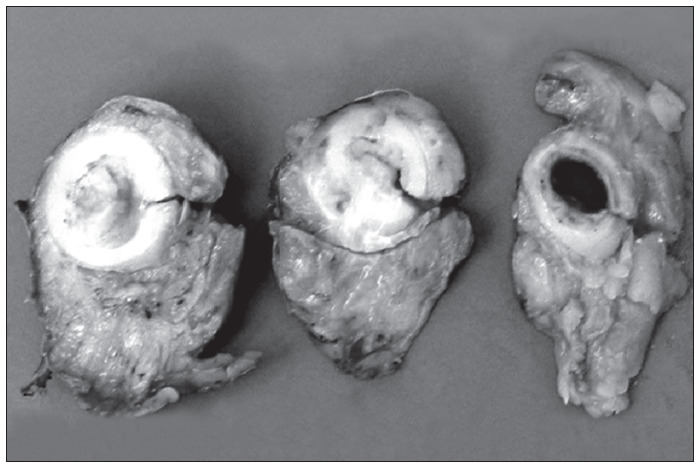
Surgical specimen of rectal endometriosis.

**Figure 4:**
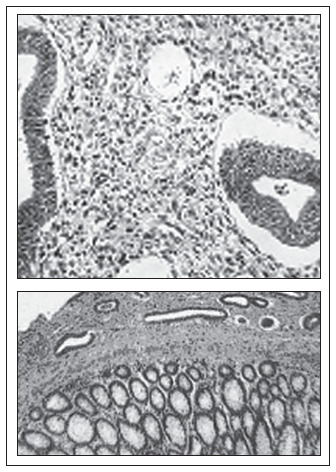
Extensive intestinal endometriosis.

**Figure 5:**
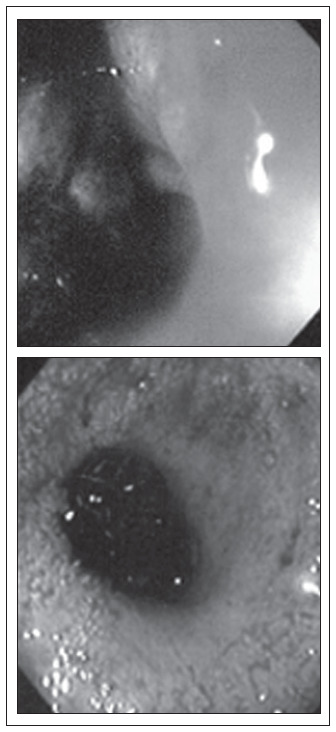
Colonoscopy in the fourth postoperative month after rectosigmoidectomy.

In view of the obstructive nature of the lesion, surgical treatment was chosen. This revealed a fibrous mass measuring 4 cm in diameter in the rectum. Abdominal rectosigmoidectomy was then performed with a low mechanical colorectal anastomosis and transversostomy in a protective loop (Figure 3). The anatomopathological examination confirmed the presence of extensive transmural intestinal endometriosis (Figure 4). The patient had a satisfactory postoperative course. Four months later, during a control colonoscopy, a wine-colored polyp was observed in the colorectal anastomosis (Figure 5) and was excised. Anatomopathological examination confirmed recurrence of the disease, which caused aggressive stenosis of the anastomosis despite new transanal resections and dilatations. The patient has remained colostomized since then, and has not been in a suitable condition for intestinal transit reconstruction.

## DISCUSSION

The rate of intestinal involvement in endometriosis cases is 3 to 34%.^8-10^ The rectum, sigmoid colon, vermiform appendix, terminal ileus and cecum are the most affected segments, in decreasing order of occurrence.^10,12^ The main symptoms are abdominal or pelvic pain, rectal pain, dysmenorrhea, dyspareunia, constipation, tenesmus and rectal bleeding. More than 90% of these patients report some type of abdominal pain, while only 20% complain of rectal bleeding.^10^ The symptoms are usually more marked during the menstrual period, but may also occur at any other time.^13^

Colorectal endometriosis is predominantly subserosal, rarely involving the muscularis or the mucosa. Colonoscopy is not always useful, but is of benefit by ruling out other lesions such as adenocarcinomas. Among the imaging examinations, endorectal ultrasound is particularly important. This has better sensitivity and specificity than computed tomography (CT) and nuclear magnetic resonance (NMR).^14^

The differential diagnoses of colonic lesions include adenocarcinomas, sarcomas, lymphomas, carcinomas and intestinal endometriosis.^5^ In cases of intestinal endometriosis, the need for colectomy ranges from 0.1 to 0.7% of the cases^8^ since the rate of endometrial carcinomas in ectopic endometrial tissue is very low. The presence of pelvic pain refractory to clinical treatment that originates in lesions of intestinal endometriosis and the obstructive nature of the disease are some of the few justifications for the procedure.^10,12,13^ In the present case, the decision to proceed with surgery was due to the stenosing nature of the lesion.

Hormonal treatment should be considered for childless young women, since this may lead to disappearance or reduction of the colorectal symptoms.^10^ Surgical treatment should be instituted when the response to conservative treatment is inadequate or there are contraindications. In selected patients, intestinal resection in combination with hysterectomy plus bilateral salpingo-oophorectomy has yielded the best results.^10^

In advanced cases with extensive pelvic and rectal involvement, fibrosis and ureteral involvement usually impair the use of surgery, with frequent need for low resections and a protective stoma. Thus, in these cases, for better control over the disease, we chose hysterectomy plus bilateral salpingo-oophorectomy in combination with rectal surgery.

The peculiar feature of the present case was the difficulty in explaining the appearance of the lesion in a patient who had been in a hypoestrogenic condition for 22 years and had undergone hysterectomy and oophorectomy three years before the present event. Moreover, she had no history suggesting the presence of endometriosis during the menacme (pelvic pain or infertility). In a case of this type, the hypothesis of intestinal tissue metaplasia should be considered.^15^

Advanced disease has been more strongly associated with pure or mixed undifferentiated disease. The results of Abrão et al. demonstrated that advanced disease with a worse outcome is related to higher prevalence of an undifferentiated pattern.^1^

## CONCLUSION

The present case cannot be explained by currently accepted theories for the etiopathogenesis of endometriosis (retrograde flow and immunological theories). Histopathologically advanced disease and more aggressive degrees of undifferentiation may explain the lower hormonal dependence with appearance of the disease during the postmenopausal period. This case emphasizes the difficulty in making a differential diagnosis with colorectal neoplasia, in view of the aggressive and recurrent nature of this disease following the menopause.
